# Proactive Control Strategies for Overt and Covert Go/NoGo Tasks: An Electrical Neuroimaging Study

**DOI:** 10.1371/journal.pone.0152188

**Published:** 2016-03-24

**Authors:** Monica Angelini, Marta Calbi, Annachiara Ferrari, Beatrice Sbriscia-Fioretti, Michele Franca, Vittorio Gallese, Maria Alessandra Umiltà

**Affiliations:** 1 Department of Neuroscience, Unit of Physiology, University of Parma, Parma, Italy; 2 Azienda Ospedaliero-Universitaria di Parma, Parma, Italy; 3 Department of Pharmacy, University of Parma, Parma, Italy; University Zurich, SWITZERLAND

## Abstract

Proactive and reactive inhibition are generally intended as mechanisms allowing the withholding or suppression of overt movements. Nonetheless, inhibition could also play a pivotal role during covert actions (i.e., potential motor acts not overtly performed, despite the activation of the motor system), such as Motor Imagery (MI). In a previous EEG study, we analyzed cerebral activities reactively triggered during two cued Go/NoGo tasks, requiring execution or withholding of overt or covert imagined actions, respectively. This study revealed activation of pre-supplementary motor area (pre-SMA) and right inferior frontal gyrus (rIFG), key nodes of the network underpinning reactive inhibition of overt responses in NoGo trials, also during MI enactment, enabling the covert nature of the imagined motor response. Taking into account possible proactive engagement of inhibitory mechanisms by cue signals, for an exhaustive interpretation of these previous findings in the present study we analyzed EEG activities elicited during the preparatory phase of our cued overt and covert Go/NoGo tasks. Our results demonstrate a substantial overlap of cerebral areas activated during proactive recruitment and subsequent reactive implementation of motor inhibition in both overt and covert actions; also, different involvement of pre-SMA and rIFG emerged, in accord with the intended type (covert or overt) of incoming motor responses. During preparation of the overt Go/NoGo task, the cue is encoded in a pragmatic mode, as it primes the possible overt motor response programs in motor and premotor cortex and, through preactivation of a pre-SMA-related decisional mechanism, it triggers a parallel preparation for successful response selection and/or inhibition during the response phase. Conversely, the preparatory strategy for the covert Go/NoGo task is centered on priming of an inhibitory mechanism in rIFG, tuned to the instructed covert modality of motor performance and instantiated during subsequent MI, which allows the imagined response to remain a potential motor act.

## Introduction

Response inhibition is the ability to suppress inadequate but automatically activated, prepotent or ongoing response tendencies (for review, see [1]). In the framework of motor inhibition, two distinct operating strategies have been described: “proactive” and “reactive” control modes [2]. In the proactive modality, inhibition or, more generally, planned action strategies are recruited in advance by predictive external or endogenous signals, and actively maintained before their enactment, in order to optimize performance in a goal-driven manner. Conversely, in the reactive control mode, inhibition is phasically enacted after the detection of the inhibitory signal.

To date, ample evidence points to a core cerebral network for reactive inhibition comprising the right inferior frontal gyrus (rIFG), the pre-supplementary motor area (pre-SMA) and the basal ganglia (BG) [2, 3]. Moreover, functional magnetic resonance imaging (fMRI) studies showed that cerebral activations during proactive and reactive inhibition largely overlap [4–8]. These findings suggest that at least part of the neural network for reactive inhibition could be recruited in advance [2], priming cortical regions in preparation for the upcoming inhibition and influencing the efficiency of inhibitory control. Nevertheless, the overlapping of proactively and reactively engaged inhibitory areas is only partial, with some brain regions selectively associated with each of these mechanisms [4, 6–8]. In this regard, it has been proposed that reactive inhibition would principally rely on rIFG, subthalamic nucleus (STN) and the hyperdirect BG circuit. Conversely, when the inhibitory goal is known in advance, proactive selective inhibition involving the dorsolateral prefrontal cortex (DLPFC) and pre-SMA, would set up inhibitory response channels in the striatum and the indirect BG loop, targeting specific thalamocortical projections to primary motor cortex and, later on, triggered by the inhibitory signal [2]. In particular, the role of the rIFG in proactive inhibition remains unclear: while some studies showed its activation in preparatory response slowing (e.g., [5]), others did not find its involvement in proactive response inhibition (e.g., [4, 6–8]).

So far, proactive and reactive inhibitory mechanisms have been investigated only during tasks in which the requested response to be stopped or withheld was an “overt” action execution (AE) (i.e., a movement effectively performed). Nevertheless, inhibitory mechanisms are also relevant for motor control during “covert actions” (i.e., potential motor acts not overtly performed), such as Motor Imagery (MI). MI is the conscious, voluntary mental rehearsal of action representations without any overt movement [9]. Previous studies revealed a substantial, even if incomplete, overlap of activated motor-related brain networks in premotor, parietal and subcortical regions [10, 11] during overtly executed and imagined movements. Nevertheless, the neural bases of motor inhibition during MI, preventing covert action from being overtly performed, in spite of the activation of the motor system, remain to be fully clarified. A first evidence in this regard comes from the results of a previous high density EEG study, in which we analyzed cerebral activities during two types of cued Go/NoGo task, requiring the execution or withholding of an overt (Go) or a covert (MI) action, respectively [12]. In that study, we showed that key nodes of the inhibitory circuit, underpinning the inhibition of the overt motor response during the NoGo condition, were also activated during the MI enactment. Indeed, in both cases inhibition relied on the activation of pre-SMA and rIFG, but with different temporal patterns of activation in accord with the intended “covert” or “overt” modality of motor performance [12]. During the NoGo condition, the pre-SMA and rIFG were sequentially activated, pointing to an early decisional role of pre-SMA and to a later role of rIFG in the enactment of inhibitory control of the overt action. Conversely, a concomitant activation of pre-SMA and rIFG emerged during the imagined motor response. This latter finding suggests that an inhibitory mechanism (likely underpinned by the rIFG), could be prewired into a prepared covert modality of motor response, as an intrinsic component of the MI enactment. This mechanism would allow the rehearsal of the imagined motor representations, without any overt movement. Of note, these data point to the involvement of proactive control strategies, suggesting that the strict cooperation between proactive and reactive mechanisms, required for successful motor control of overt actions, could be also relevant in the covert motor context.

In the present study, to test this hypothesis, we analyzed our previous cued overt and covert Go/NoGo EEG data sets, focusing on cerebral activities time-locked to the preparatory signals. We investigated whether the overt and covert modality of the possible incoming motor response, instructed by the preparatory cue, predisposed *ab initio* a different organization of the parietofrontal areas involved with sensorimotor transformations and motor inhibitory control. To this purpose, we performed a spatiotemporal analysis of the cue-elicited scalp electric fields [13]. This approach avoids the methodological limitations of the “canonical” waveform analysis, which usually selects scalp sites and time periods to evaluate amplitude and latency of predefined event related potential (ERP) components of interest [13]. Moreover, the spatiotemporal analysis method allows one to distinguish whether simple modulation in response strength or, conversely, dissimilar scalp electric field topographies underpin different elicited EEG activities between conditions. Hence, this approach provides a more objective definition of time windows for source analysis, relying on the statistical proof that the electric fields are different and thus produced by different neural generators [13].

## Materials and Methods

### Participants

Fifteen young adult volunteers took part in the study: nine male, six female; mean age 24.4 years (standard deviation, *SD* = 3.81); age range: 20–35 years. All participants had normal or corrected-to-normal visual acuity, no history of psychiatric or neurological impairments and were right-handed, as assessed by the Edinburgh Handedness Inventory [14]. All participants provided a written informed consent to participate in the study, which has been approved by the local ethical committee (Comitato Etico per Parma, Azienda Ospedaliero-Universitaria di Parma, Azienda Unità Sanitaria Locale di Parma, Università degli Studi di Parma) and has been conducted according to the principles expressed in the Declaration of Helsinki.

### Stimuli and Procedure

**Fig 1:**
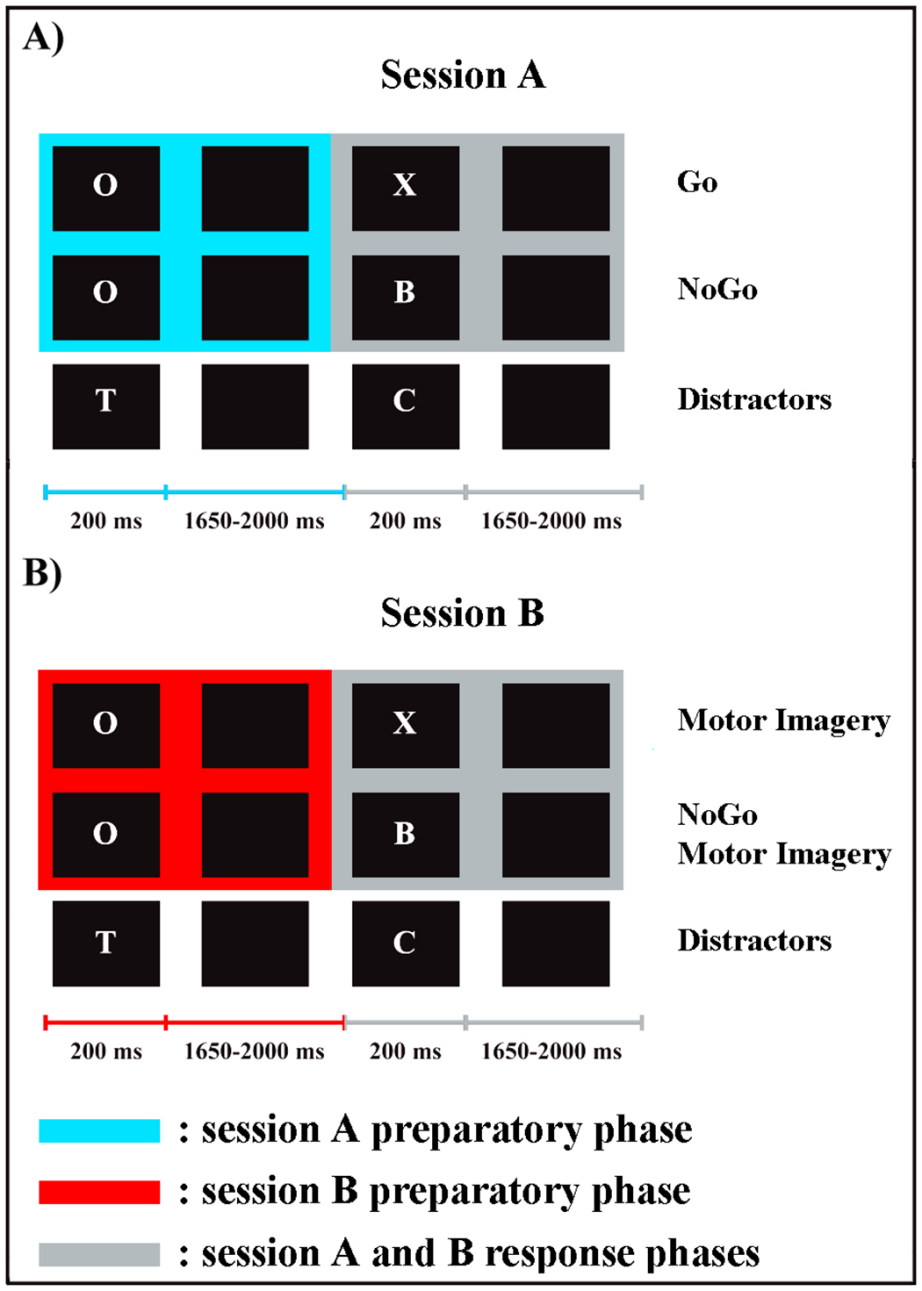
Experimental paradigm and stimuli. The present study analyzed the preparatory phases of sessions A and B (for the analyses of the response phases of both sessions, see [12]).

The experimental paradigm and stimuli have been detailed elsewhere [12]. In brief, we used a modified form of a cued Continuous Performance Task (CPT) [15], consisting of four experimental conditions organized in two blocks (sessions A, B) (Fig 1A and 1B): overt Go and NoGo conditions were presented in session A (overt Go/NoGo task); MI and NoGo Motor Imagery (NoGoMI) conditions were tested in session B (covert Go/NoGo task). The order of presentation of the two sessions was balanced among participants. Stimuli consisted of 12 different white letters (*A-H*, *J*, *L*, *O* and *X*) on a black background, sequentially presented in pseudo-random order at the center of a 19-inches computer screen positioned at 60 cm from participants. The same letter was never immediately repeated. Each letter, presented for 200 ms, was separated from the next one by a black screen whose duration varied randomly between 1650 and 2000 ms. In both sessions (Fig 1) the letter “O” was the preparatory cue, representing a warning signal to prepare to respond. It was followed by the imperative target stimulus, which specified the requested response. Hence, each trial of the four conditions consisted in a preparatory phase (between the onset of the cue letter “O” and the onset of the successive target stimulus) and in a response phase (between target onset and the onset of the successive letter). The present study is focused on the preparatory phase of the two sessions (for results and discussion about the response phase, see [12]). In session A (Fig 1A) the letter “X” after the “O” cue represented the target stimulus for Go condition. It required the overt execution of a motor response, consisting in pressing a button on a pad, positioned in front of the participants, with the index finger of the right hand. In session B (Fig 1B) the letter “X” after the “O” cue represented the target stimulus for the MI condition, requesting the participants to perform a kinesthetic MI of the button-press movement (i.e., to imagine themselves pressing the button in a first-person perspective). In both sessions the other letters (*A*–*H*, *J*, *L*) required response inhibition if they were preceded by an “O”, representing target stimuli in session A for NoGo and in session B for NoGoMI conditions, respectively; if not preceded by an “O”, they served as meaningless distractors. Each of the two sessions consisted of 80 trials “O-X” (Go and MI trials, in session A and B, respectively), 80 trials “O-noX” (NoGo and NoGoMI, in session A and B, respectively) and 240 distractors. The sequence of presentation of trials and distractors was randomized. Each session lasted about 20 minutes, with a five minutes rest period between the two sessions.

Stimuli delivery and response recording were controlled using E-prime 2.0 software; the button-press recording was used to assess omission (i.e., Go trials without responses) and commission (i.e., responses in NoGo trials) errors.

### EEG Recording and Preprocessing

Continuous EEG was recorded using the 128-channels Geodesic EEG System (Electrical Geodesics Inc., Oregon) and the HydroCel Geodesic Sensor Net (GSN300), at a sampling rate of 500 Hz (0.01 Hz high-pass filter) with the vertex as on-line reference; electrodes impedances were kept below 50 kΩ. Off-line analyses were performed with Cartool software [16]. The raw EEG data were band-pass filtered (1–30 Hz) and recalculated against the average reference.

To evaluate cue-elicited ERPs, epochs from 200 ms before to 1000 ms after “O” letter onset were averaged across trials, separately for each participant and session; these single-subject averages were then used to compute two group-averaged ERPs, one for each experimental session. Trials with erroneous responses (omission and commission errors), NoGo, MI and NoGoMI trials with concomitant EMG activity during the response phase, as well as trials with EMG activity before target onset were excluded (see EMG recording). The remaining trials were submitted to an automated threshold rejection criterion of 65 μV and visually inspected for detection of ocular, muscular and other artifacts. To maintain a good signal-to-noise ratio, a lower limit of 80 artifact-free correct trials per participant per session was set. The mean of accepted trials was 95.13 (*SD* = 11.2) for session A and 92.13 (*SD* = 10.2) for session B. A two-tailed *t*-test (*p* < .05) was performed in order to exclude differences in the number of accepted trials between sessions, which did not result significant (*t* = 1.36, *p* = .26). The outermost belt of electrodes of the sensor net, more prone to show residual muscular artifacts, was excluded and the original template was reduced from 128 to 110 channels. Artifacted channels were interpolated using a spherical spline interpolation method implemented in Cartool software [17].

For completeness, we obtained the ERPs also for distractors, in both experimental sessions: these additional analyses are reported in S1 File and S1 Fig.

### EEG Analysis

EEG data were subjected to two independent analysis procedures. The first one, a global ERP waveform analysis, was performed for completeness and as a preliminary step in determining the time course of ERP response modulations, to minimize the possibility of missed effects related to the preselection of specific electrodes and time periods used in canonical ERP waveform analysis.

The second one was a global scalp electric field analysis. We based the present study on this latter type of analysis, because it has two important advantages: 1) it is completely reference independent; 2) it allows the statistical assessment of the likely neurophysiological mechanisms (i.e., topographic and/or strength modulation) underpinning the observed effects [13].

All the statistical analyses were conducted using Cartool software [16].

#### 1) Global ERP waveform analysis

**Fig 2:**
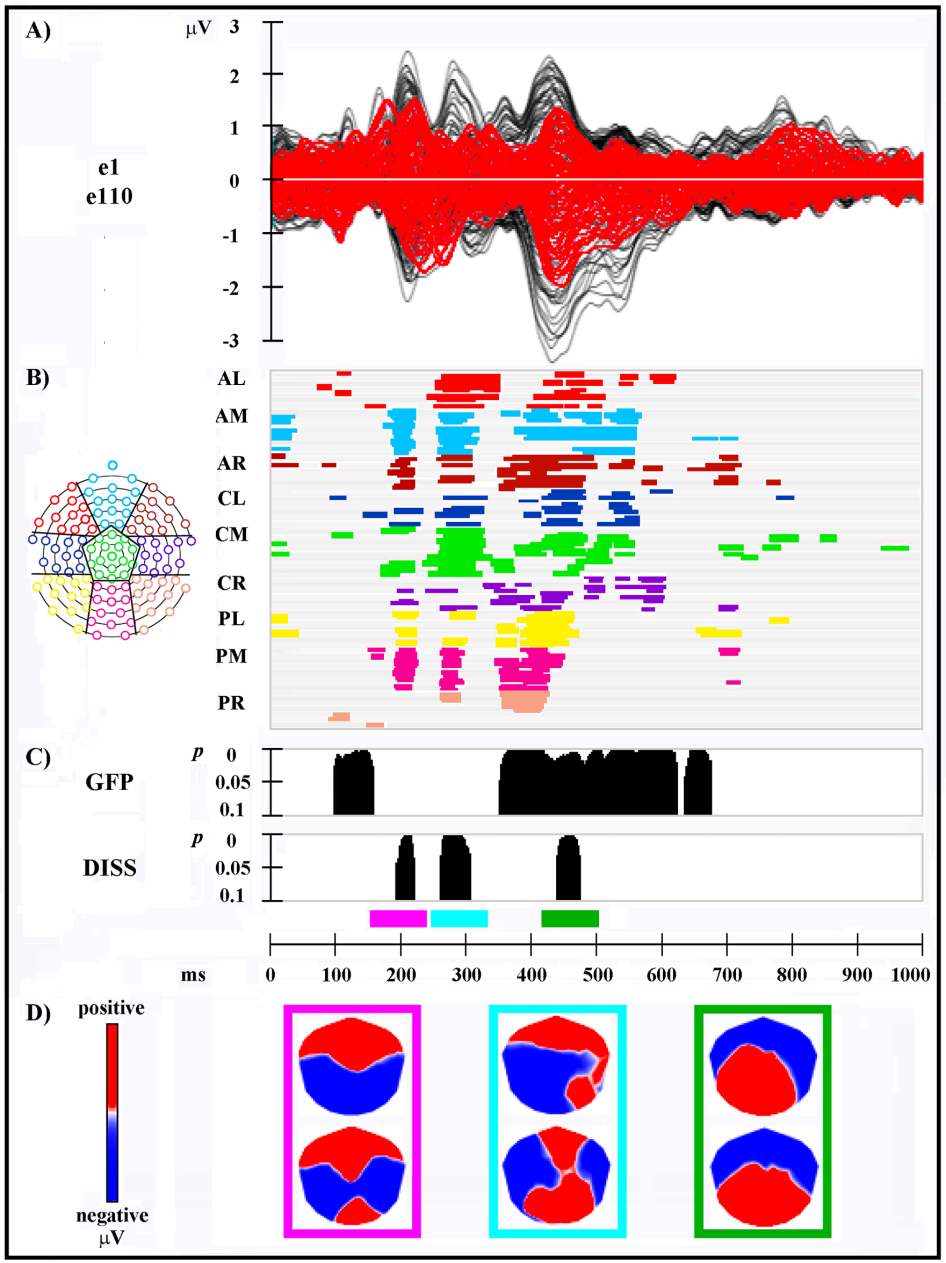
Electrophysiological results over 1000 ms after cue onset (cue onset: 0 ms). **(A)** Group-averaged (n = 15) event related potential (ERP) waveforms of the two experimental sessions, superimposed across the 110 recording channels (e1–e110). Black: session A cue; red: session B cue. **(B)** Statistical analysis of global ERP amplitude. Periods of significant differences of ERP amplitude (*p* < .05; duration ≥ 20 ms) at each electrode and time point between sessions are displayed as colored horizontal lines. Each horizontal line represents one scalp electrode. Different colors indicate different clusters of electrodes; the distribution of the clusters over the electrode montage is shown in the inset on the left side of the figure. AL: anterior left; AM: anterior midline; AR: anterior right. CL: central left; CM: central midline; CR: central right. PL: posterior left; PM: posterior midline; PR: posterior right. **(C)** Global scalp electric field analyses. Upper plot: statistical analysis of global electric field strength. Black areas indicate time intervals of significant differences (*p* < .05; duration ≥ 20 ms) of Global Field Power (GFP) between sessions. Lower plot: statistical analysis of global electric field topography (topographic analysis of variance, TANOVA). Black areas indicate time intervals of significant differences (*p* < .05; duration ≥ 20 ms) of global spatial dissimilarity index (DISS) between sessions. **(D)** Mean topographic maps of the group-averaged ERP data for session A (upper map) and session B (lower map) cues, corresponding to each time interval of significant topographic modulation between sessions resulting from TANOVA. Each panel is colored as the corresponding TANOVA time interval. All topographic maps are plotted with nasion upward and left scalp leftward; each map is scaled separately with respect to its maximum and minimum values to optimise the contrast.

The global waveform analysis was conducted by means of point-wise paired *t*-tests computed on amplitudes of the single-subject ERP averages of the two sessions, at each electrode and time point. The statistical significance level was set at *p* < .05 and a 10 contiguous data points temporal criterion (20 ms at our 500 Hz sampling rate) for the persistence of significant effects was applied [18]. Only differences over at least five contiguous electrodes within nine clusters (shown in the inset in Fig 2) reaching the statistical significance level were retained.

#### 2) Global electric field analyses

Two statistical analyses were conducted on the global electric field: a) assessment of modulations in electric field strength, as measured by the instantaneous Global Field Power (GFP); b) assessment of modulations in electric field topography, measuring the global spatial dissimilarity index (DISS) [19].

Significant modulations in GFP and DISS between the experimental sessions were assessed by non-parametric statistical analyses based on point-wise randomization tests [20]. Randomization provides a robust non-parametric method to test for differences in any variable without any assumption regarding data distribution, by comparing the observed data set with random shuffling of the same values over sufficiently large number of iterations (i.e., permutations); this method allows one to determine the probability that the data might be observed by chance. In the present study, the point-wise randomization tests ran 1000 permutations per data point and the significance level was set at *p* < .05, with an additional temporal stability acceptance criterion of 20 ms of consecutive significant difference [18].

These two analyses allowed a neurophysiological interpretation of the ERP modulations: indeed, differences in GFP without simultaneous topographic changes are indicative of amplitude modulation of statistically indistinguishable generators between experimental conditions. Conversely, topographic differences between conditions, with or without concomitant GFP modulations, necessarily derive from changes in the configuration of the underlying active brain sources [13].

Changes in electric field strength were assessed by means of the statistical comparison of the GFP between sessions for each participant [13, 19]. GFP is the spatial standard deviation of the potentials at all electrodes at a given time point: it is calculated as the square root of the mean of the squared value recorded at each electrode (measured versus the average reference) and has higher values for stronger electric fields [13, 19]. Point-wise paired randomizations were conducted on the GFP of single-subjects ERP averages between sessions at each time frame, with a significance level set at *p* < .05 and a temporal acceptance criterion of 20 ms of consecutive significant difference.

Significant periods of topographic modulation were identified using randomization statistics applied to DISS [13, 19] between sessions, calculated for each time point and each participant data. DISS is a strength-independent index of configuration differences between two electric fields and it is calculated as the square root of the mean of the squared differences between the instantaneous voltage potentials (measured versus the average reference) across the electrodes montage, each of which is first scaled to unitary strength by dividing it by the instantaneous GFP. Point-wise paired randomizations were performed on the DISS data: this analysis is also known as “topographic analysis of variance” (TANOVA) [13]. As above, 1000 permutations for each time point were performed and only effects with *p* < .05 and lasting for 20 ms or longer [18] were considered significant.

While GFP modulations indicate quantitative changes, DISS modulations between sessions reflect qualitative changes in the underlying generators configuration [13].

### Source Analysis

The results of the above topographic global scalp electric field analysis (TANOVA) defined time periods during which intracranial sources were estimated, using a distributed linear inverse solution based on a Local Auto-Regressive Average (LAURA) regularization approach [21]. LAURA model reconstructs the brain electric activity in each point of a 3D grid of solution points, selecting the source configuration that better mimics the biophysical behavior of electric fields without *a priori* assumption on the number of dipoles in the brain. The solution space was calculated on a locally spherical head model with anatomical constraints (L-SMAC) [22] and comprised 3001 solution points (voxels) homogeneously distributed within the brain structures of the Montreal Neurological Institute (MNI152) average brain. All solution points were labeled with their Talairach and Tournoux coordinates [23] as well as their anatomical labels.

Intracranial source estimations for each participant and session over time windows defined by the TANOVA were then statistically compared by means of a “voxel-wise parametric mapping analysis” [24]. To do that, individual ERP data were averaged over time periods of significant topographic modulation, in order to generate a single data point per period for each participant and session. The LAURA current density source estimations for each solution point were then contrasted by means of paired *t*-tests. Solution points with *p* values < .05 (*t*_(14)_ > 2.14/ < -2.14) were considered significant; in addition, a cluster threshold of at least 10 contiguous activated solution points was applied. Source analyses were performed using Cartool software [16].

### EMG Recording and Analysis

During both experimental sessions, surface EMG of the first dorsal interosseous muscle of the participant’s right hand was recorded continuously with EGI’s Polygraph Input Box (PIB) (sampling rate 500 Hz, band-pass filter 30–200 Hz, notch 50 Hz). EMG recording aimed at excluding the presence of movements or muscular twitches during the preparatory phase of all trials and during the response phase in NoGo, MI and NoGoMI trials. The computed procedure of EMG analysis for trials rejection has been detailed elsewhere [12].

## Results

The electrophysiological results of global amplitude and scalp electric field analyses and the results of source analyses are reported separately. Significant results of the statistical comparisons of LAURA source estimations (voxel-wise parametric mapping analysis) in significant TANOVA time periods are reported, with *t* and *p* values, Talairach and Tournoux coordinates (x,y,z) [23] and anatomical labels of solution points with the local maximum different activities.

### Electrophysiological Results

**Fig 3:**
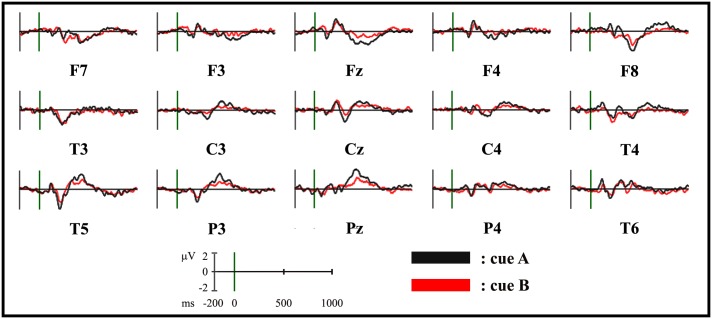
ERP waveforms for the cues in the two experimental sessions at selected electrodes. Group-averaged (n = 15) cue-locked ERP waveforms, plotted as voltage in μV in function of time in ms (stimulus onset: 0 ms). Black: session A cue; red: session B cue.

The electrophysiological results of global amplitude and scalp electric field analyses are shown in Fig 2. The group-averaged ERPs for session A and session B cues, superimposed across the 110 recording channels (e1–e110) and at selected electrodes, are shown in Figs 2A and 3, respectively.

The global amplitude analysis (Fig 2B) revealed three periods of significant ERP modulation:

1) from 188 to 222 ms after cue onset, over anterior clusters of electrodes, on a right and midline location, and over posterior clusters of electrodes, at a left and midline location; 2) from 246 to 324 ms after cue onset, in all scalp regions; 3) from 346–558 ms after cue onset, in particular over posterior clusters of electrodes from 352 to 458 ms, and over frontal and central clusters of electrodes during the whole time window.

The analysis of the GFP (Fig 2C, upper plot) showed three periods of sustained difference between sessions, reflecting a strength modulation with a stronger activity in session A: 1) from 95 to 158 ms; 2) from 350 to 622 ms; 3) from 632 to 674 ms after cue onset.

The TANOVA (Fig 2C, lower plot) revealed three phases of significant topographic differences between sessions, reflecting the activation of distinct configurations of intracranial generators: 1) from 192 to 220 ms; 2) from 258 to 306 ms; 3) from 438 to 472 ms after cue onset. Mean topographic maps of the group-averaged ERP data of session A and B, corresponding to each significant TANOVA time interval, are shown in Fig 2D.

In summary (Fig 2), GFP and DISS analyses revealed that different cerebral generators underpinned the first (between about 190–220 ms after cue onset) and the second (between about 245–325 ms) periods of amplitude modulation between sessions, which overlapped with periods of significant different scalp field topography. The third prolonged amplitude modulation (between 345–560 ms after cue onset) was characterized by both strength and topographic differences between sessions.

### Source estimations

**Fig 4:**
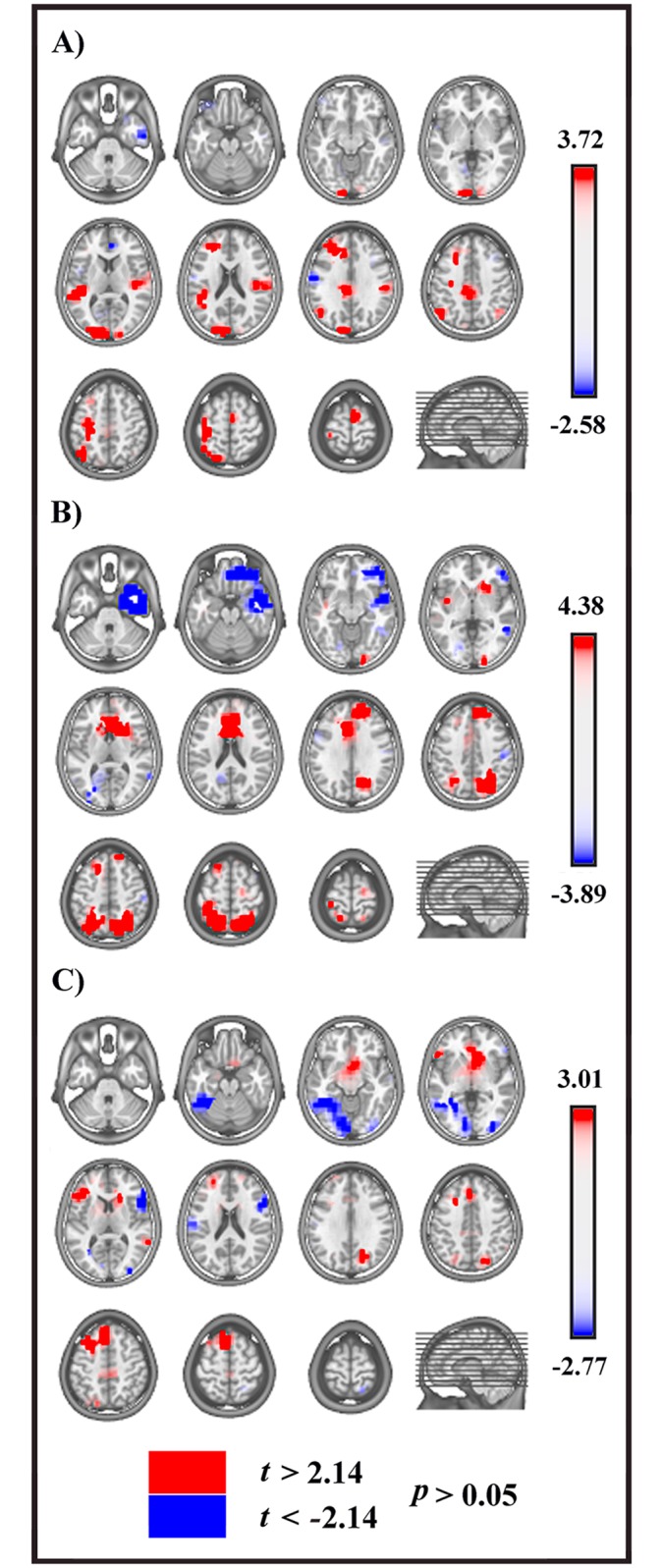
Statistical comparisons of LAURA source estimations between session A and B over significant TANOVA time intervals. All significant voxels are colored (*t*_(14)_ > 2.14 / < -2.14, *p* < .05): positive *t* values (red color) indicate higher current source densities in session A than in session B; negative *t* values (blue color) indicate higher current source densities in session B than in session A. LAURA solutions are rendered on MNI152 template brain (left hemisphere on the left side). **(A)** First significant TANOVA time interval (192–220 ms after cue onset). **(B)** Second significant TANOVA time interval (258–306 ms after cue onset). **(C)** Third significant TANOVA time interval (438–472 ms after cue onset).

For the first time period of different topography (192–220 ms after cue onset) significant higher activity in session A as compared with session B (Fig 4A, red) was found in four cerebral clusters: 1) left precentral and postcentral gyri, extending toward middle frontal gyrus (BAs 3, 4, 6) (*t*_(14)_ = 3.72; *p* = .002; x,y,z: -33,-16,46; left precentral gyrus, BA 4); 2) left occipital extrastriate cortex, encompassing cuneus and middle occipital gyrus (BA 18) (*t*_(14)_ = 3.71, *p* = .002; x,y,z: -18,-90,31; left occipital cuneus, BA 18); 3) left superior temporal gyrus (BA 22) (t _(14)_ = 3.37, *p* = .005; x,y,z: -48,-39,12; left superior temporal gyrus, BA 22); 4) left prefrontal cortex, encompassing DLPFC and frontopolar cortex (BAs 9, 10) (*t*_(14)_ = 2.88, *p* = .012; x,y,z: -18,27,29; left medial frontal gyrus, BA 9).

In the second significant TANOVA period (258–306 ms after cue onset) higher activity in session A (Fig 4B, red) was found in three clusters: 1) bilateral posterior parietal cortex (PPC), encompassing superior parietal lobule (SPL) and precuneus (BA 7) (*t*_(14)_ = 4.38, *p* = .0006; x,y,z: 26,-53,40; right SPL, BA 7); 2) bilateral dorsal anterior cingulate cortex (dACC) (*t*_(14)_ = 2.83, *p* = .013; x,y,z: -11,19,23; anterior cingulate cortex, BA 24); 3) right DLPFC, encompassing superior and medial frontal gyri (BA 9) (*t*_(14)_ = 2.76, *p* = .015; x,y,z: 11,41,29; right medial frontal gyrus, BA 9).

Higher activity in session B (Fig 4B, blue) was found in two clusters: 1) right anterior temporal and temporopolar cortex, encompassing superior, middle and inferior temporal gyri (BAs 20, 21, 38) (*t*_(14)_ = -3.89, *p* = .002; x,y,z: 48,1,-35; right middle temporal gyrus, BA 21); 2) right orbitofrontal cortex, encompassing rectal and middle frontal gyri (BA 11) (*t*_(14)_ = -3.56, *p* = .003; x,y,z: 11,39,-24; right rectal gyrus, BA 11).

In the third period of topographic modulation (438–472 ms after cue onset) significant higher activation in session A (Fig 4C, red) was found in two cerebral clusters: 1) left pre-SMA (BA 6) (*t*_(14)_ = 2.88, *p* = .012; x,y,z: -3,21,58; left superior frontal gyrus, BA 6); 2) left ventrolateral prefrontal cortex (VLPFC) (BAs 45, 47) (*t*_(14)_ = 2.68, *p* = .018; x,y,z: -56,25,2; left IFG, BA 45).

Higher activity in session B (Fig 4C, blue) was found in two cerebral clusters: 1) rIFG (BA 45) (*t*_(14)_ = -2.77, *p* = .015; x,y,z: 56,18,9; rIFG, BA 45); 2) left fusiform gyrus (BA 37) *(t*_(14)_ = -2.33, *p* = .035; x,y,z: -33,-48,-13; left fusiform gyrus, BA 37).

## Discussion

The aim of the present study was to evaluate whether different response strategies were triggered in the preparatory phase of two different cued Go/NoGo tasks, requiring the execution or inhibition of an overt motor response (AE) in session A and of a covert motor response (MI) in session B, respectively. In particular, we aimed to clarify whether the preparatory cues proactively elicited putatively similar inhibitory circuits that appeared to be subsequently involved in the withholding of the overt response in NoGo trials and in the inhibition of overt movement execution during the MI trials [12].

Our results confirmed in both sessions the priming of cerebral regions pertaining to such putative inhibitory network reactively triggered in the following response phase; nonetheless, difference in the preparatory strategies between the two sessions emerged, depending on the intended “overt” or “covert” performance modality of the possible incoming motor response.

The TANOVA analysis showed that topographic differences in preparatory activities between the two sessions started around 190–220 ms after the cue onset. In this time period, the higher activation in session A of a motor region encompassing the left motor and dorsal premotor cortex (dPMC) (Fig 4A), revealed by source analysis, could index the cue-elicited priming of the incoming overt Go-response representations. This result is consistent with evidence that motor programs can be automatically triggered by visual warning signals [25, 26], preparing the motor system for the execution of the selected response at subsequent target onset. Nevertheless, to prevent wrong cue-triggered overt movements, a concomitant inhibitory mechanism is required. In this regard, it has been hypothesized that premotor cortex could encode both response-specific motor programs and their concomitant automatic inhibition [27]: the activation of the left dPMC that emerged in our results is consistent with this proposed “impulse control” mechanism [27], activated during response preparation and aimed to maintain in check the selected response until imperative signals onset. Furthermore, in our overt Go/NoGo task, the activation of the left dPMC emerged not only during the preparatory phase, as showed in the present results, but also in the following response phase during NoGo trials [12]. Altogether, these findings are in line with previous evidence pointing to the role of the dPMC in both proactive [28] and reactive motor inhibition [29].

It is worth noting that in the present paradigm the cue did not specify the identity of incoming targets, acting as a generic warning signal and introducing a conflict between representations of the possible forthcoming responses. Indeed, in the second TANOVA time period, higher activity in session A emerged in cerebral areas that have been related to conflict processing, namely the right PPC and the dACC (Fig 4B). It has been proposed that the right PPC might be relevant in situations of conflict between action plans, and in particular in the presence of competition between stimulus-driven action representations and the voluntary control of behavior ([30]; for review see [31]). Moreover, in agreement with the “conflict monitoring hypothesis” [32], the dACC would provide an online signal between contrasting cue-elicited Go and NoGo task goal representations, recruiting cerebral areas for the resolution of such conflict and the selection of appropriate response. In this regard, it has been hypothesized that the pivotal function of the pre-SMA in motor preparation and control is the resolution of conflict within a contingent set of competitive response plans [33, 34], and that its putative role in response inhibition could represent a particular instantiation of this general function [35]. Accordingly, a higher activation of the pre-SMA emerged in session A in the following third TANOVA time interval (Fig 4C). Moreover, taking into account the whole overt Go/NoGo task, the pre-SMA engagement emerged not only during the preparatory phase, but also in the following response phase, with higher activity during NoGo with respect to Go trials [12]. These findings are consistent with a previous human intracranial recording study, which found pre-SMA activation during the preparatory period and later on, just before movement inhibition [5]. Our data point to the involvement of the pre-SMA in both proactive and reactive inhibition of overt actions. Nevertheless, the exact role of the pre-SMA in proactive inhibition remains unclear: this area could set up the nodes of the inhibitory network in advance, favoring their speeded activation when inhibition is needed [5]. The pre-SMA could also participate in the modulation of the threshold of Go response and of the level of excitability of the motor system [36, 37] in the presence of conflicting instructions, or when inhibition is a possible response option. Regardless of the specific mechanism involved, the pre-SMA seems to exert inhibitory control within a network including the rIFG and the BG [2, 3]. Although the rIFG has been claimed as the crucial area for the actual implementation of motor inhibition [2, 3], to date its role in proactive control is still debated. Our analyses did not reveal the activation of the rIFG in the preparation of the overt Go/NoGo task in session A; conversely, activation of the rIFG emerged during the subsequent NoGo response [12]. Taken together, our results sustain the involvement of the rIFG in reactive inhibitory control of overt actions, in agreement with previous studies that did not find the pre-engagement of the rIFG but its activation just during the implementation of inhibition [4, 6, 7]. Hence, the recruitment of the inhibitory circuit during the preparation of the overt Go/NoGo task was only partial, and limited to the decisional function underpinned by pre-SMA.

On the contrary, the engagement of the rIFG emerged during the preparation of the covert Go/NoGo task in the third TANOVA interval (Fig 4C). In session B participants knew that their incoming performance would have been limited to covert movements: since they were not requested to overtly move during the whole session B, theoretically there was neither need to activate an inhibitory mechanism in response to the cue, nor to postulate a cue-triggered anticipation of the NoGoMI signal. According to our hypothesis, this finding likely represents the cue-elicited strategy of inhibition of motor outputs in anticipation of the possible incoming imagined covert response. The priming of the rIFG would allow the subsequent automatic enactment of an inhibitory mechanism during MI [12], assuring the covert nature of the imagined motor response. Hence, the preparatory strategy for the covert Go/NoGo task was focused on a prioritized recruitment of inhibition of motor outputs in anticipation of the possible incoming imagined motor inhibition of the covert response, tuned to the task motor goal requiring just a covert action. Conversely, and differently from the overt Go/NoGo task, during session B the cue-triggered preactivation of the motor or premotor cortex did not emerge. A possible explanation could be related to the fact that in the present paradigm the cue did not specify the identity of subsequent targets: the uncertainty created by such generic cue, about whether or not the MI would have been requested by the incoming target, could have abolished the functional advantage of priming the motor representations for the covert response. In the light of these considerations, future studies comparing overt and covert Go/NoGo tasks using a completely informative cue (i.e., a cue that is associated with a single target and response option) could confirm and further extend our results concerning proactive inhibitory and motor strategies for overt and covert actions. Finally, due to the low spatial accuracy of EEG technique, further neuroimaging studies with higher spatial resolution should better clarify the selective involvement of different sectors of complex regions as the PPC and the IFG, and the contribution of subcortical structures such as the BG, the STN or the cerebellum, in the preparation of inhibitory processing in overt and covert actions.

## Conclusions

The results of the present study demonstrate a substantial overlap of cerebral networks activated during proactive recruitment and subsequent reactive enactment of motor inhibition in both overt and covert actions; at the same time our data show a differential involvement of the pre-SMA and rIFG, in accord with the intended type of covert or overt of incoming motor response.

During the preparation of the overt Go/NoGo task, the cue is encoded in a pragmatic mode: it primes the possible overt motor response programs in motor and premotor cortex and, through preactivation of a pre-SMA-related decisional mechanism, it triggers a parallel preparation for the successful response selection and/or inhibition during the response phase. Conversely, the preparatory strategy for the covert Go/NoGo task is centered on the goal-oriented priming of an inhibitory mechanism related to the rIFG that, being tuned to the instructed covert modality of the motor performance and instantiated during the subsequent MI enactment, allowed the imagined response to remain a potential motor act. Hence, during the preparatory phase of our cued overt and covert Go/NoGo tasks, the different adopted strategies are tuned to the “how” of the motor performance, reflecting the intended overt and covert modality of the possible incoming action.

## Supporting Information

S1 File Supplementary Materials. Additional information about distractors-related ERPs in the two experimental sessions.
(DOCX)

S1 Fig Supplementary Figure 1. Event related potential (ERP) waveforms for cues and distractors in the two experimental sessions, plotted as voltage in μV in function of time in ms (stimulus onset: 0 ms). **(A)** Upper plot: group-averaged (n = 15) ERP waveforms for session A cue and distractors, superimposed across the 110 recording channels (e1–e110). Lower plot: ERPs at selected electrodes for session A cue and distractors. Black: cue; green: distractors. **(B)** Upper plot: group-averaged (n = 15) ERP waveforms for session B cue and distractors, superimposed across the 110 recording channels (e1–e110). Lower plot: ERPs at selected electrodes for session B cue and distractors. Red: cue; blue: distractors.
(TIF)
