# Correction: Proactive Control Strategies for Overt and Covert Go/NoGo Tasks: an Electrical Neuroimaging Study

**DOI:** 10.1371/journal.pone.0155007

**Published:** 2016-05-02

**Authors:** Monica Angelini, Marta Calbi, Annachiara Ferrari, Beatrice Sbriscia-Fioretti, Michele Franca, Vittorio Gallese, Maria Alessandra Umiltà

The Data Availability statement for this paper is incorrect. The correct statement is: All relevant data are within the paper, its Supporting Information files and from Zenodo: http://dx.doi.org/10.5281/zenodo.49173
