# Supplementary material for: Proactive Control Strategies for Overt and Covert Go/NoGo Tasks: An Electrical Neuroimaging Study
Source: PLoS One. 2016 Mar 24;11(3):e0152188. doi: 10.1371/journal.pone.0152188 (PMC4807103; doi:10.1371/journal.pone.0152188)
Supplement: S1 File — Additional information about distractors-related ERPs in the two experimental sessions. (DOCX) [file pone.0152188.s001.docx]

**Supplementary Materials**

For completeness, in addition to the ERPs evoked by the cues, we also evaluated the ERPs related to the distractors in both experimental sessions.

**Supplementary Methods**

To evaluate distractors-elicited ERPs, epochs from 200 ms before to 1000 ms after distractor letters onset were averaged across trials, separately for each participant and session; these single-subject averages were then used to compute two group-averaged distractors ERPs, one for each experimental session. Only distractor trials without overt wrong responses or concomitant EMG activity were retained. The accepted trials were submitted to an automated threshold rejection criterion of 65 μV and visually inspected for detection of ocular, muscular and other artifacts. The outermost belt of electrodes of the sensor net was excluded and the original template was reduced from 128 to 110 channels. Artifacted channels were interpolated using a spherical spline interpolation method implemented in Cartool software.

Since in our overt and covert cued CPT Go/NoGo tasks the number of distractors was higher than the number of cues (see “Experimental Paradigm”), we randomly selected a subset of the accepted distractor epochs, in order to average a similar number of cues and distractors trials to obtain the respective ERPs. The mean ± SD of accepted trials for the distractors was: 95.5 ± 8.7 for session A; 89.2 ± 6.36 for session B. An ANOVA confirmed that the number of accepted trials were not significantly different among cues and distractors (F _(3,42)_: 1.65, *p*: .19).

**Supplementary Results and Discussion**

The ERPs for cues and distractors in the two experimental sessions are shown in S1 Fig.

By visual inspection, the comparison between grand-averaged ERPs clearly evidences different brain processing for cue and for distractors. In both sessions, the distractors evoked a N1 potential at posterior temporoparietal electrodes, with higher amplitude with respect to the cue (amplitude, latency and location of maximal negative deflection over the selected electrodes shown in S1 Fig. A and B, lower panels: for session A cue: -2.32 µV, 208 ms in T5; for session A distractors -4.19 µV, 180 ms in T5; for session B cue -1.47 µV, 224 ms in T5; for session B distractors: -3.54 µV at 180 ms in T6). Later on, a higher positive deflection with a maximum in parietal electrodes in P3 time range was present in cue-related ERPs (amplitude, latency and location of maximal positive deflection over the selected electrodes shown in S1 Fig. A and B, lower panels: for session A cue: 2.37 µV, 440 ms in Pz; for session A distractors 1.12 µV, 464 ms in T5; for session B cue 1.4 µV, 452 ms in Pz; for session B distractors 0.87 µV, 456 ms in T5). These findings could be interpreted in the framework of attentive mechanisms and visual discrimination processes. Indeed, the comparison between cue- and distractor-related electrophysiological activities elicited during the CPT is used to assess attention-related mechanisms, such as the perceptual ability to discriminate between task relevant and irrelevant stimuli and the subsequent selection of behavioral-relevant cue stimuli for further processing (for a review on CPTs, see Supplementary Reference [1]). During visual discrimination processes, the early posterior N1 could reflect orienting toward stimuli that are not only salient, but also relevant to the ongoing task, showing modulation by top-down mechanisms. Moreover, the selection of specific task relevant stimuli for their further encoding is thought to be indexed by later ERPs in particular in P3 time range [2, 3]. Accordingly, in our data the higher amplitude of posterior N1 for distractors and the presence of a higher parietal P3 for the cues, suggest that attention might initially be captured and then withdrawn from stimuli that had to be ignored [4]. Of note, in our cued CPT Go/NoGo tasks two factors have to be taken in to account when considering the attentional effect of distractors [5, 6, 7]: 1) their relevance for the ongoing task (i.e., the effect of the “task contingency” of distractors stimuli); 2) the influence of different levels of sensory and working memory load between cues and distractors. Regarding the “task contingency” of distractors, in our paradigm the same letters (*A-H, J, L)* were indeed both distractors if not preceded by an “O*”* letter, and targets for action withholding if presented after the “O” cue. This goal-related feature of distractors can potentially act as a confounding factor, requiring a higher level of attention during their discrimination. Another relevant aspect is that while the cue in both sessions is represented by a single letter (*O*), distractors are represented by 10 different letters (*A-H, J, L*), hence requiring a discrimination process that could affect the N1 amplitude [7]. In the present study, both these features could have determined an easier detection of the cue, and a concomitant higher perceptual and attentional demand for the discrimination and recognition of the distractors. This enhanced cognitive and attentional load during the processing of the distractors could be functional for their faster identification: since distractor stimuli do not require further responses, this mechanism could permit the disengagement of cognitive resources and their reallocation to the analysis of the following stimuli.

**Supplementary References**

1 Riccio CA, Reynolds CR, Lowe P, Moore JJ. The continuous performance test: a window on the neural substrates for attention? Arch Clin Neuropsychol. 2002;17:235-272.

2 Herrmann CS, Knight RT. Mechanisms of human attention: event-related potentials and oscillations. Neurosci Biobehav Rev. 2001;25:465-476.

3 Brandeis D, Banaschewski T, Baving L, Georgiewa P, Blanz B, Warnke A, et al. Multicenter P300 brain mapping of impaired attention to cues in hyperkinetic children. J Am Acad Child Adolesc Psychiatry. 2002;41:990-998.

4 Kiss M, Grubert A, Eimer M. Top-down task sets for combined features: behavioral and electrophysiological evidence for two stages in attentional object selection. Atten. Percept. Psychophys. 2013;75:216-228.

5 Lavie N. Distracted and confused?: selective attention under load. Trends Cogn Sci. 2005; 9: 75-82.

6 Fellrath J, Manuel AL, Ptak R. Task relevance effects in electrophysiological brain activity: early, but not first. Neuroimage. 2014;101:68-75.

7 Vogel EK, Luck SJ. The visual N1 component as an index of a discrimination process. Psychophysiology. 2000;37:190-203.
