# Supplementary figures and images for: Proactive Control Strategies for Overt and Covert Go/NoGo Tasks: An Electrical Neuroimaging Study
Source: PLoS One. 2016 Mar 24;11(3):e0152188. doi: 10.1371/journal.pone.0152188 (PMC4807103; doi:10.1371/journal.pone.0152188)

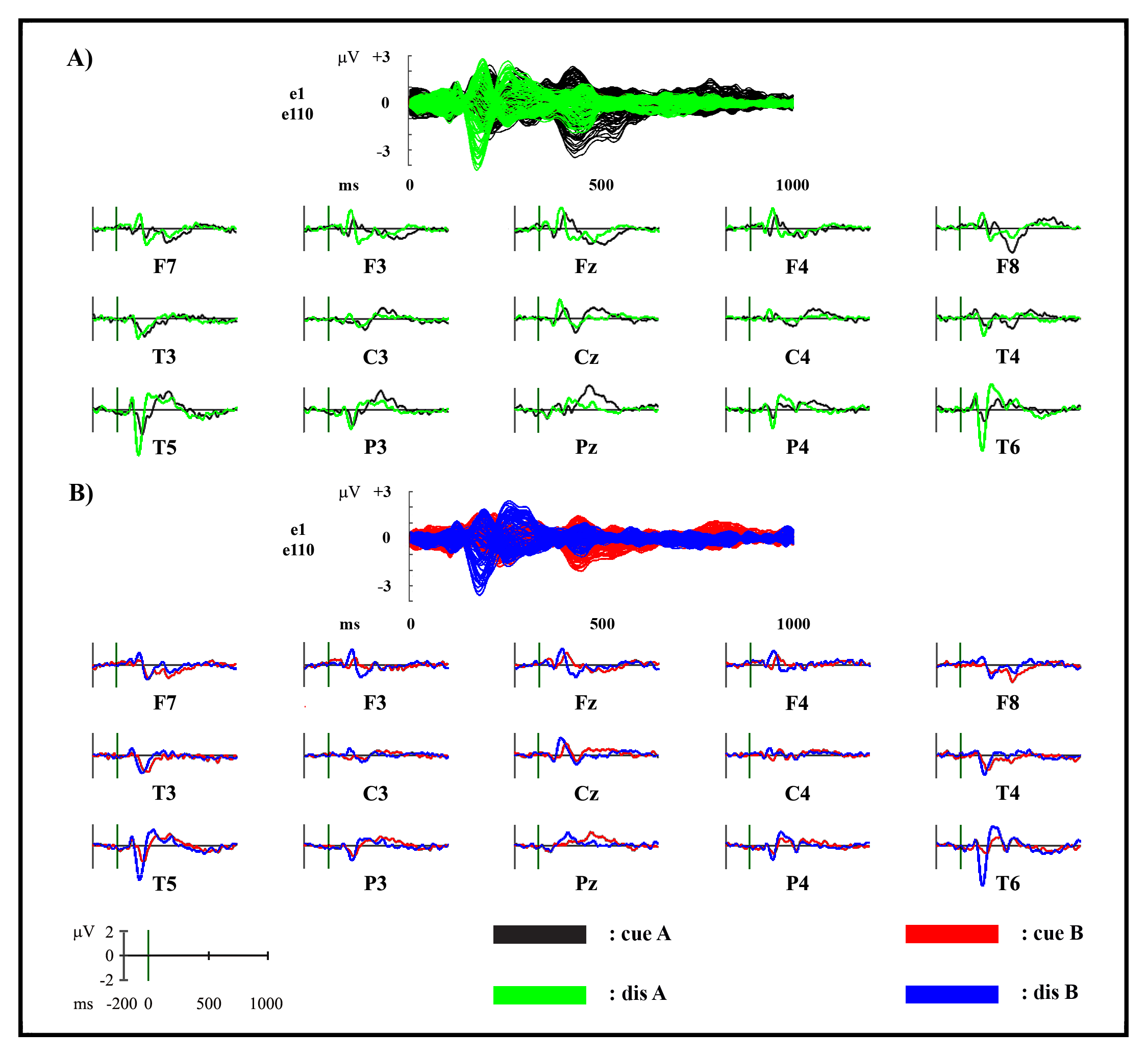

Supplement: S1 Fig — Event related potential (ERP) waveforms for cues and distractors in the two experimental sessions, plotted as voltage in μV in function of time in ms (stimulus onset: 0 ms). (A) Upper plot: group-averaged (n = 15) ERP waveforms for session A cue and distractors, superimposed across the 110 recording channels (e1–e110). Lower plot: ERPs at selected electrodes for session A cue and distractors. Black: cue; green: distractors. (B) Upper plot: group-averaged (n = 15) ERP waveforms for session B cue and distractors, superimposed across the 110 recording channels (e1–e110). Lower plot: ERPs at selected electrodes for session B cue and distractors. Red: cue; blue: distractors. (TIF) [file pone.0152188.s002.tif]
